# Design of substrate-based BCR-ABL kinase inhibitors using the cyclotide scaffold

**DOI:** 10.1038/srep12974

**Published:** 2015-08-12

**Authors:** Yen-Hua Huang, Sónia T. Henriques, Conan K. Wang, Louise Thorstholm, Norelle L. Daly, Quentin Kaas, David J. Craik

**Affiliations:** 1 Institute for Molecular Bioscience, The University of Queensland, Brisbane, Queensland, 4072, Australia

## Abstract

The constitutively active tyrosine kinase BCR-ABL is the underlying cause of chronic myeloid leukemia (CML). Current CML treatments rely on the long-term use of tyrosine kinase inhibitors (TKIs), which target the ATP binding site of BCR-ABL. Over the course of treatment, 20–30% of CML patients develop TKI resistance, which is commonly attributed to point mutations in the drug-binding region. We design a new class of peptide inhibitors that target the substrate-binding site of BCR-ABL by grafting sequences derived from abltide, the optimal substrate of Abl kinase, onto a cell-penetrating cyclotide MCoTI-II. Three grafted cyclotides show significant Abl kinase inhibition *in vitro* in the low micromolar range using a novel kinase inhibition assay. Our work also demonstrates that a reengineered MCoTI-II with abltide sequences grafted in both loop 1 and 6 inhibits the activity of [T315I]Abl *in vitro*, a mutant Abl kinase harboring the “gatekeeper” mutation which is notorious for being multidrug resistant. Results from serum stability and cell internalization studies confirm that the MCoTI-II scaffold provides enzymatic stability and cell-penetrating properties to the lead molecules. Taken together, our study highlights that reengineered cyclotides incorporating abltide-derived sequences are promising substrate-competitive inhibitors for Abl kinase and the T315I mutant.

Chronic myeloid leukemia (CML) is a hematopoietic disease characterized by increased and unregulated growth of predominantly myeloid cells. From onset, CML typically progresses through three clinical phases: the chronic phase, the accelerated phase, and the terminal blastic phase. Philadelphia chromosome (Ph), the cytogenetic hallmark of CML, results from the reciprocal translocation of chromosomes 9 and 22. This fusion between breakpoint cluster (BCR) gene from chromosome 22 and Abelson (ABL) tyrosine kinase gene from chromosome 9^1^,^2^ forms the *BCR-ABL* oncogene. The *BCR-ABL* gene encodes persistently high levels of cytoplasmic and constitutively active BCR-ABL tyrosine kinase, which is detected in >90% of CML patients and 25% of adult patients with acute lymphocytic leukemia (ALL)^3^. Animal studies also provide evidence to support BCR-ABL as the oncogenic cause of CML as it was shown to induce a myeloproliferative syndrome that closely resembles the chronic phase of human CML^4^.

Philadelphia chromosome-positive (Ph^+^) patients in chronic phase of CML rely on sustained administration of small-molecule tyrosine kinase inhibitors (TKIs). The first-line therapy is imatinib mesylate (IM, also known as STI-571 or Gleevec^®^), a TKI that binds to the ATP cleft of the inactive form of BCR-ABL and prevents the conformational change required for kinase activation^5^. Clinical resistance to TKI therapy is a significant issue in the treatment of CML patients in the advanced stage of the disease^1^,^6^, primarily because the induction of point mutations in the BCR-ABL kinase domain impair the interaction between IM and the ATP binding cleft^7^. Two second generation TKIs, dasatinib^8^,^9^ and nilotinib^9^, and one third generation TKI, bosutinib^10^,^11^,^12^, were developed to overcome IM-resistant BCR-ABL mutants; however, none have shown significant activity against T315I—the most problematic of the mutants due to its resistance to multiple TKIs. In 2012, ponatinib^13^ (AP24534, Iclusig™) was approved by the Food and Drug Administration (FDA) as a therapeutic for CML or ALL Ph^+^ patients carrying the T315I mutation. Although ponatinib has shown potent inhibition against all clinically important BCR-ABL single mutants including T315I, compound mutants harboring the T315I mutation are highly resistant to this TKI^13^,^14^,^15^. Therefore, overcoming BCR-ABL-dependent resistance to current CML therapies remains a major challenge in drug design.

In addition to the ATP cleft, the catalytic domain of BCR-ABL (Fig. 1a) includes a second distinct site: a substrate-binding site. Kinase substrates have larger contact area with the kinase domain than ATP, and the substrate-binding site is specific to each kinase, suggesting that inhibitors targeting this site would be less affected by mutations compared to TKIs^16^. Thus, peptide inhibitors targeting the substrate-binding site are an alternative strategy that can be used to inhibit BCR-ABL with higher specificity than the small molecule TKIs.

Substrate-based kinase inhibitors are typically designed using knowledge on a range of peptide substrates^17^,^18^. A large study of kinase specificity using 2.5 billion synthetic peptides and nine tyrosine kinases^19^,^20^ led to the identification of the consensus motif Ile/Val/Leu-Tyr-Xaa-Xaa-Pro/Phe (where Xaa is any amino acid) required for substrate recognition by Abl kinase. As Abl kinase shares the same feature of the catalytic domain of BCR-ABL that is crucial for its oncogenetic activities, abltide (EAIYAAPFAKKK), the optimal substrate of Abl kinase containing the consensus motif, can be used as a starting point for a rational design of a substrate-based inhibitor of the oncogenic BCR-ABL.

Although peptides have high target specificity and low toxicity profiles, their development as therapeutics is hampered by their low stability and limited access to intracellular space^21^. The discovery of cyclotides, peptides that display a head-to-tail cyclic backbone and a cystine knot motif (Fig. 1b), which imparts high stability against physical and enzymatic degradations^22^, has opened new doors for the development of peptide-based drug candidates. Some cyclotides, such as MCoTI-I and MCoTI-II (*Momordica cochinchinensis* trypsin inhibitor-I and II)^23^, have also been reported to penetrate cells^24^,^25^ (and have subsequently been classified as cyclic cell-penetrating peptides^26^), suggesting that they can also be used to deliver bioactive peptides into cells. Indeed, they have been used successfully as scaffolds for the design of stable peptide therapeutics with non-native bioactivities and/or for intracellular delivery^27^,^28^,^29^,^30^,^31^,^32^. Examples include: a bioactive sequence active against the 3C protease of the foot-and-mouth-disease virus grafted into loop 1 of MCoTI-II^32^, potent pro-angiogenic peptides inserted into loop 6 of MCoTI-II^28^, and the introduction of the N-terminal fragment of p53 into loop 6 of MCoTI-I, resulting in an anti-tumor compound^31^.

In the current study MCoTI-II was chosen as a framework to stabilize linear peptide sequences active against Abl kinase and to deliver them into cells. With this approach we developed novel peptide-based inhibitors with prolonged serum stability and cell-penetrating properties that target the substrate-binding site of BCR-ABL and potentially prevent the binding of the endogenous substrates.

## Results

Eight MTAbl peptides with foreign sequences grafted into loop 6 or into loop 1 of MCoTI-II were designed using molecular modeling. Six of these peptides and several variants designed to increase cellular uptake were synthesized. Inhibitory activity against native Abl kinase and the [T315I]Abl mutant, as well as serum stability, cytotoxicity and cellular uptake of the lead compounds were evaluated.

### Molecular modeling of grafted MCoTI-II containing the abltide sequence bound to Abl kinase

A molecular model of the linear abltide bound to Abl kinase was first constructed to facilitate the design of grafted MCoTI-II analogs. This model was built by homology with the crystal structures of an abltide variant bound to an inactive form of Abl kinase^5^ and of an active form of protein kinase A bound to a peptide substrate, ATP and two magnesium ions (PDB ID: 1atp). Molecular dynamics (MD) simulations of the model were stable over 40 ns, and the backbone root mean square deviation (rmsd) to the crystal structure conformation was only ~2 Å (Supplementary Fig. S1). The conformation of abltide remained almost identical to that in the crystal structure of modified abltide, with a backbone rmsd of ~1.5 Å.

The MCoTI-II scaffold was then “docked/grafted” into the Abl/abltide complex using abltide as an anchor, producing models of peptides MTAbl00–03 and 08–11 in complex with Abl kinase. Each system was studied using triplicate 20 ns MD simulations, and the resulting binding poses suggested that the tested MTAbl peptides mainly interact with the C-lobe of Abl kinase (Supplementary Fig. S2). A total of four MTAbl peptides grafted in loop 6 and four MTAbl peptides grafted in loop 1 were considered for molecular modeling, as detailed below.

The cyclic peptides with grafts in loop 6 were designed by replacing the native loop with the first nine residues of abltide (MTAbl00) and with one additional glycine residue at the N-terminus (MTAbl01), C-terminus (MTAbl03), or at both termini (MTAbl02). The stability of the grafted sequences was evaluated using the backbone rmsd of the grafted peptide (graft rmsd) from its conformation in the linear abltide/Abl complex after fitting the complex structures on the kinase backbone. The graft rmsd of MTAbl02 was ~1.5 Å, as shown in Fig. 2, comparable to the backbone rmsd measured during simulations of Abl in complex with linear abltide (Supplementary Fig. S3). Therefore, the simulations suggest that abltide grafted in MTAbl02 can adopt a conformation that is nearly identical to that displayed by linear abltide when in complex with Abl. In contrast, the simulations of three other MTAbl peptides showed that the conformation of their grafted abltide diverged from that of linear abltide, with graft rmsd values of ~2.0 Å, ~2.5 Å and ~3.0–4.0 Å for MTAbl00, MTAbl01 and MTAbl03, respectively (Fig. 2). The models suggest that the C-terminus of grafted abltide sequences is located in a region of negatively charged potential generated by Abl kinase. Consequently, several grafted analogs in loop 6, MTAbl04, MTAbl05, MTAbl06 and MTAbl07, were designed to incorporate additional basic residues at the C-terminus of the abltide sequence.

The engineering of MCoTI-II grafted in loop 1 initially focused on optimizing the length of the graft because loop 1 of MCoTI-II has only six residues whereas linear abltide is 12 residues. The designed peptides incorporated the first seven (MTAbl08), eight (MTAbl09) or ten (MTAbl10) residues of abltide into the scaffold. A nine-residue graft comprising the first eight residues of abltide preceded by a glycine residue (MTAbl11) was also considered. The molecular modeling studies of grafts in loop 6 indicated that adding a glycine at the C-terminus was detrimental to binding, and the addition of a spacer at the N-terminus of the grafted peptide in MTAbl11 could allow the peptide to adopt a conformation compatible with binding at the interface. Results from the MD simulations suggested that the grafted segment of MTAbl09 adopts a conformation that is almost identical to that of linear abltide at the interface (graft rmsd ~1.5 Å). The conformation of grafted segments in the three other peptides, MTAbl08, MTAbl10 and MTAbl11, deviated from the optimal abltide conformation, as shown by their graft rmsds reaching 2.5 Å or above (Supplementary Fig. S4). The simulations of MTAbl08 were found to be particularly unstable, leading to very high graft rmsd values. The three simulations of MTAbl09 converged toward a similar binding mode, but those of MTAbl10 and MTAbl11 did not, suggesting that the scaffold does not form strong or specific interactions with the kinase.

### Synthesis and structure of grafted peptides

**Figure 1:**
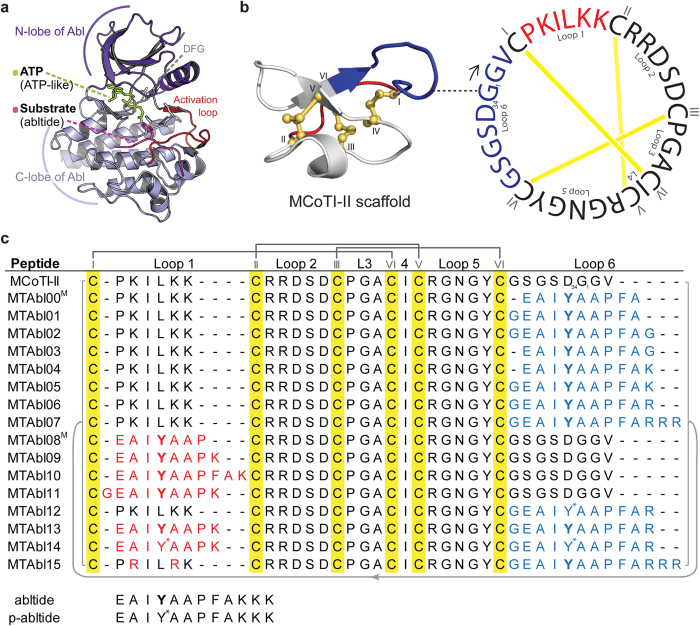
Three-dimensional structures of Abl kinase and MCoTI-II, and amino acid sequences of MCoTI-II variants considered in this study. (**a**) Abl kinase with substrate-ATP conjugate bound to the catalytic site (PDB ID: 2g2f). The substrate (abltide, in magenta) binds in the cleft between the N- and C-lobes; the phosphorylation site is oriented towards the ATP binding pocket in the N-lobe. (**b**) Three-dimensional structure and amino acid sequence of native MCoTI-II (PDB ID: lib9). The cysteine-rich peptide has a unique cyclic cystine knot (CCK) motif, comprising a cyclic backbone and three interlocking disulfides (shown in yellow). The starting point of the peptide sequence (G_1_) is connected to the corresponding position on its ribbon structure with a dashed line. The six cysteine residues partition the backbone into six loops. Loops 1 and 6, which were replaced with foreign sequences in this study, are highlighted in red and blue, respectively. (**c**) Sequence alignment of native MCoTI-II and MTAbl peptides. The six cysteines are highlighted in yellow and numbered using Roman numerals (I–VI). Foreign sequences containing the recognition motif of Abl kinase inserted into loops 1 or 6 are colored in red and blue, respectively. The phosphorylatable tyrosines are in bold font and the phosphorylated tyrosine residues are labeled with an asterisk. The Cys I–IV, II–V and III–VI disulfide linkages are shown using dark gray lines. MCoTI-II and all the MTAbl peptides are head-to-tail cyclized, indicated by a light gray line. The affinity of MTAbl00 and MTAbl08 to Abl kinase was evaluated using molecular modeling only (labeled with a superscript M).

Fourteen mutant peptides (MTAbl01-07, 09-15) with abltide analogs grafted in loop 1, loop 6 or both loops 1 and 6 were synthesized (Fig. 1c). The one-dimensional ^1^H NMR spectra of all MTAbl peptides showed well dispersed peaks in the amide region and aligned well with the spectrum of MCoTI-II, suggesting that these peptides adopt native-like folds (data not shown). Two-dimensional homonuclear NMR was used to determine the Hα chemical shifts of three representative MTAbl peptides, namely MTAbl07, MTAbl09 and MTAbl13. A comparison of secondary Hα chemical shifts of these grafted peptides to MCoTI-II is shown in Fig. 3. The secondary Hα chemical shifts of the scaffold positions (gray-shaded region in Fig. 3) were comparable to the equivalent positions of MCoTI-II, suggesting that the three-dimensional structures of MTAbl07, MTAbl09 and MTAbl13 are similar to the parent peptide MCoTI-II. As expected, the main differences in Hα chemical shifts between MCoTI-II and MTAbl mutants were observed in the mutated loops (*i.e*. loop 1 and/or loop 6) or the regions near these two loops.

### Abl kinase inhibition by peptide-based inhibitors

The kinase inhibitory effect of the grafted peptides was assessed using the BacKin assay^33^, which quantifies phosphorylation of abltide displayed on the surface of *E. coli* cells. The percentage of abltide phosphorylated by Abl kinase is measured using flow cytometry after incubation with biotinylated anti-phosphotyrosine antibody and subsequent labeling with streptavidin-conjugated phycoerythrin. Unlike canonical kinase inhibition methods, for example ADP-Glo™ kinase assay (Promega), which evaluates the kinase activity by quantifying the amount of ADP formed from a kinase reaction, BacKin assay offers an alternative way to examine the kinase inhibitory activity by quantifying the level of phosphorylated substrates on a solid support. This novel assay was developed for the evaluation of inhibitory activities of kinase substrates, which cannot be fairly evaluated using ADP-Glo™.

Abltide has inhibitory activity at low micromolar concentrations (IC_50_: 5.7 μM) against Abl kinase, as measured using the BacKin assay, and, interestingly, phosphorylated abltide (p-abltide) has a similar activity. This similarity suggests that abltide, either non-phosphorylated or phosphorylated, can bind to Abl kinase and inhibit its activity. The importance of the phosphorylatable tyrosine was further investigated by synthesizing a suite of 17 abltide analogs in which the tyrosine was replaced by an alanine, a phenylalanine or one of 15 unnatural phenylalanine residues (see Supplementary Fig. S5). None of these peptides demonstrated significant inhibition of Abl kinase at concentrations up to 64 μM; therefore, a tyrosine residue at position 4 of abltide seemed to be optimal for binding and for inhibition of Abl kinase. Moreover, the results indicated that the phosphorylated substrate can also act as inhibitor, suggesting that abltide could serve as an inhibitor of Abl kinase without substituting its phosphorylatable tyrosine.

**Figure 2:**
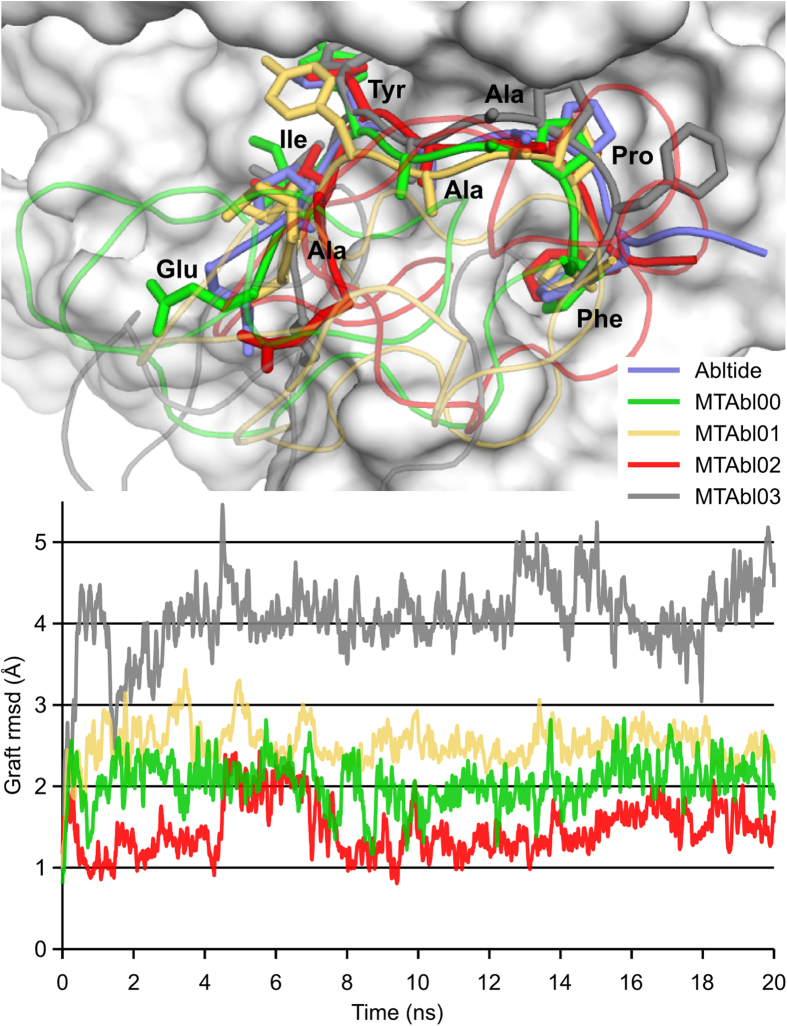
Molecular modeling of the interactions between Abl kinase and MTAbl00, MTAbl01, MTAbl02 and MTAbl03 peptides, which contain grafts in loop 6. Upper panel: Superimposition of the binding modes observed after 20 ns of molecular dynamics (MD) simulations for the four MTAbl peptides and after 50 ns MD simulations for abltide. The five MD frames were fitted onto the kinase backbone. The molecular surface of Abl kinase is shown in white. The grafted peptides and linear abltide are shown using ribbon representation and their side chains are shown using stick representations. The backbones of MTAbl peptides are shown as curvy lines. Lower panel: Evolution of the root mean square deviation of the grafted abltide (graft rmsd) over the MD simulations of the MTAbl peptides in complex with Abl kinase. The graft rmsd is computed as the backbone rmsd between the grafted abltides and the corresponding sequences in abltide after fitting the backbone of Abl kinase.

The Abl kinase inhibitory activity of MCoTI-II loop 6 variants MTAbl01–03 (Fig. 1c) was evaluated using the BacKin assay at 32 μM peptide concentration (Fig. 4a). MTAbl01, MTAbl02 and MTAbl03 inhibited 75%, 90% and 50% of kinase activity, respectively. These values correlated with the similar conformations of the grafted segments to that of the linear abltide bound to Abl kinase, as shown by molecular modeling studies (Fig. 2); therefore, the conformation of the grafted segments at the interface seems to affect activity.

Based on our initial screening and molecular modeling, three analogs, MTAbl04, MTAbl05 and MTAbl06, designed to have better charge complementarity with Abl kinase, were synthesized and tested for inhibitory activity. MTAbl04 was derived from MTAbl03 by substitution of the C-terminal glycine residue in loop 6 with a lysine residue, whereas MTAbl05 and MTAbl06 were derived from MTAbl02 by substitution of the C-terminal glycine residue in loop 6 with a lysine or arginine residue, respectively. MTAbl04 showed similar activity to MTAbl03, inducing ~50% reduction of kinase activity, whereas MTAbl05 and MTAbl06 had similar activity to MTAbl02, inhibiting ~90% of kinase activity. Therefore, the addition of one basic residue at the C-terminus did not cause a dramatic difference in activity at 32 μM peptide concentration. To appreciate subtle differences between the variants, inhibition dose response curves were evaluated and the IC_50_ values were calculated (Supplementary Table S1). MTAbl05 and MTAbl06 differed by only the C-terminal residue of loop 6, *i.e*. lysine and arginine residues, respectively, but had a 3-fold difference in activity (IC_50_: 15.2 ± 7.0 μM vs 5.5 ± 1.6 μM). To evaluate the contribution of additional positive charges in loop 6 to kinase inhibition, the dose-dependent activity of MTAbl07, which has three arginine residues at the C-terminus of loop 6, was also evaluated. MTAbl07 had comparable activity (IC_50_: 4.1 μM) to MTAbl06, indicating that extra arginine residues in loop 6 do not significantly improve binding to Abl kinase. Overall, additional basic residues at the C-terminus of loop 6 only led to small improvements in activity.

Of three loop 1 variants (MTAbl09–11), only MTAbl09 achieved 90% inhibition at 32 μM, which is equivalent to the activity of the best inhibitors with a graft in loop 6, *i.e*. MTAbl02, MTAbl05 and MTAbl06. This result is in agreement with our molecular modeling studies suggesting that MTAbl09 is the only analog with abltide grafted in loop 1 able to completely maintain the conformation adopted from linear abltide at the interface with Abl kinase. The incorporation of recognition motif in both loops 1 and 6 (MTAbl13) improved the inhibitory activity, with the IC_50_ decreasing to 1.3 μM compared to the equivalent peptides modified only in loop 1 (MTAbl09, IC_50_: 18.3 μM) or loop 6 (MTAbl06, IC_50_: 5.5 μM). Although MTAbl13 was found to be less potent than IM (IC_50_: 0.3 μM), its potency was comparable to other substrate-based kinase inhibitors from previous studies^34^,^35^. Finally, activity of MTAbl12 and MTAbl14 – the phosphorylated versions of MTAbl06 and MTAbl13, respectively – were evaluated and were found to have comparable IC_50_ values to the non-phosphorylated peptides. This result was in agreement with the measurement of inhibitory activity of phosphorylated abltide, suggesting that cyclic peptides incorporating the non-phosphorylated abltide can function as kinase inhibitors.

Ph^+^ CML patients harboring the T315I mutation in BCR-ABL do not respond to most drugs currently available. Therefore, the inhibitory activity of MTAbl peptides against [T315I]Abl kinase is of the utmost relevance and was investigated *in vitro*. The catalytic activity of [T315I]Abl kinase was first examined by comparing the phosphorylation of 30 μM abltide catalyzed by 0.2 U/mL wild-type or [T315I]Abl kinase using LC-MS. The time required to phosphorylate half of the abltide molecules by wild-type and [T315I]Abl kinase was 24.2 ± 0.7 and 50.0 ± 1.6 min, respectively (Supplementary Fig. S6). The dose-response curves of IM, MTAbl13 and its phosphorylated version MTAbl14 against wild-type Abl kinase are shown in Fig. 4b. The IC_50_ values of IM, MTAbl13 and MTAbl14 were 0.3, 1.3 and 2.6 μM, respectively. The inhibition activity of [T315I]Abl kinase by MTAbl13 was 12.2 μM, whereas IM showed no inhibitory activity up to 256 μM (Fig. 4b and Supplementary Table S1).

### MTAbl peptides are remarkably stable in human serum

The proteolytic stability of MTAbl06, MTAbl07, MTAbl09, MTAbl12 and MTAbl13 in 100% human serum was evaluated, as well as native MCoTI-II and abltide for up to 24 h (Fig. 5). All of the tested MTAbl peptides, with the exception of MTAbl12, were stable, with >75% of peptide remaining after 24 h of incubation. The lower stability observed for MTAbl12 was due to dephosphorylation of the p-Tyr residue, as suggested by RP-HPLC profile and mass spectrometry analysis in which the retention time and mass of the degradation product was the same as for MTAbl06 (i.e. dephosphorylated MTAbl12). MTAbl09 and MTAbl13 are statistically more stable than MCoTI-II according to a one-way ANOVA test (*p* < 0.05 and *p* < 0.001, respectively), whereas MTAbl07 was as stable as MCoTI-II. In contrast to the cyclic peptides, linear abltide was completely degraded in human serum within 1 h. Comparison with control samples in PBS solution showed that the binding of tested compounds to serum proteins or Eppendorf Tubes® was negligible (<5%).

### Cellular uptake of substrate-based inhibitors

Since BCR-ABL localizes predominantly in the cytoplasm, it is important to evaluate whether the designed peptides can be internalized into cells. MTAbl06, MTAbl07, MTAbl09, MTAbl13, and MTAbl15 were fluorescently labeled with one AlexaFluor® 488 molecule (A488) and their cell-penetrating properties were compared to that of labeled MCoTI-II using flow cytometry^26^,^36^. TAT, a well-studied cell-penetrating peptide^37^, was included as a positive control. Initially, we confirmed that none of these peptides displayed cytotoxicity towards HeLa cells at concentrations up to 64 μM for 2 h in a resazurin-based cell viability assay (data not shown). HeLa cells were then treated with A488-labeled MTAbl peptides at five concentrations for 1 h individually and the mean fluorescence emission intensity of treated cells measured at 530/30 nm using flow cytometry is shown in Fig. 6a. The Alexa-labeled peptides entered cells in a dose-dependent manner and the level of uptake efficiency was: TAT > MTAbl15 > MTAbl07 > MTAbl06 ≥ MCoTI-II. A comparison of peptide uptake relative to A488-MCoTI-II at 8 μM is shown in Fig. 6b. A488-MTAbl07 and A488-MTAbl15 showed statistically higher cellular uptake than A488-MCoTI-II (*p* < 0.05 and *p* < 0.001, respectively). Conversely, A488-MTAbl09 and A488-MTAbl13 showed a 25% and 10% decrease in cellular uptake, respectively, compared to MCoTI-II at the same concentration. No significant decrease in fluorescence intensity was observed after addition of trypan blue, a non-permeable quencher able to quench the fluorescence of non-internalized/membrane-bound peptides (Fig. 6b), indicating that the monitored fluorescence was not from membrane-bound but from internalized peptides.

### Solution structure of the double-grafted analog, MTAbl13

**Figure 3:**
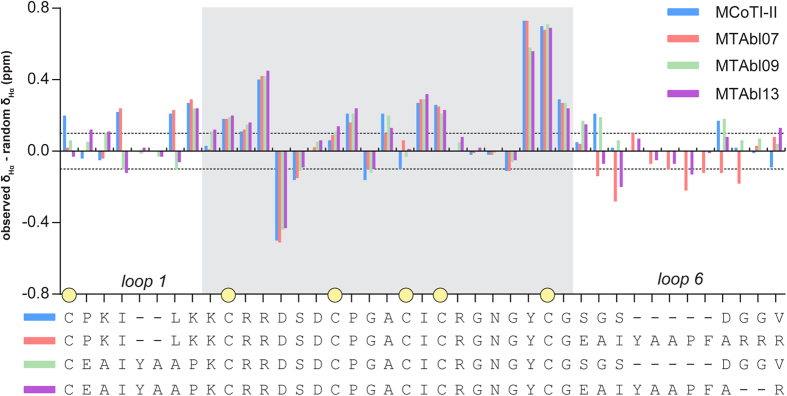
Secondary Hα chemical shift analysis of MCoTI-II and selected grafted analogs, MTAbl07, MTAbl09 and MTAbl13. The color used for each peptide is shown in the key at the top right. The dotted lines represent secondary chemical shift values of −0.1 and 0.1 ppm. A sequence alignment of the four peptides is shown below the chart. Secondary Hα chemical shifts of the region with identical sequences are shaded in gray. Yellow circles mark the positions of cysteine residues.

**Figure 4:**
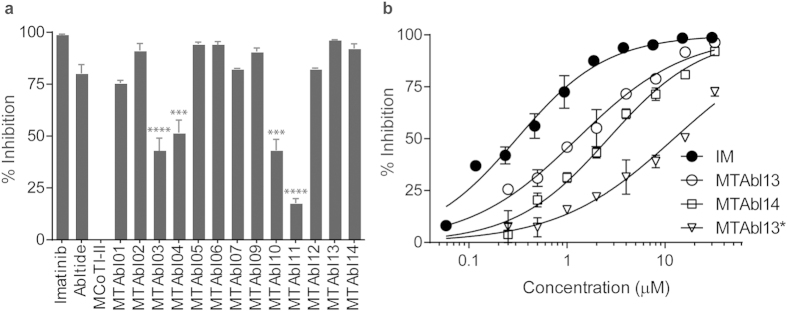
Inhibition of Abl kinase activity induced by MTAbl peptides determined using the BacKin assay. (**a**) The percentage of Abl kinase (0.2 U/mL) inhibition in the presence of 32 μM of each peptide at 37 °C for 30 min. MTAbl peptides that have significant difference in inhibition efficacy compared to abltide are marked with asterisks (****p < 0.0001; ***p < 0.001, evaluated with one-way ANOVA). (**b**) Inhibition of Abl kinase activity induced by increasing concentrations of IM, MTAbl13 or MTAbl14. MTAbl13* corresponds to inhibition of [T315I]Abl kinase. Data were fitted to a sigmoidal dose-response curve with variable slope model and analyzed with GraphPad Prism 6. Results shown here are the mean ± SEM from two independent experiments.

**Figure 5:**
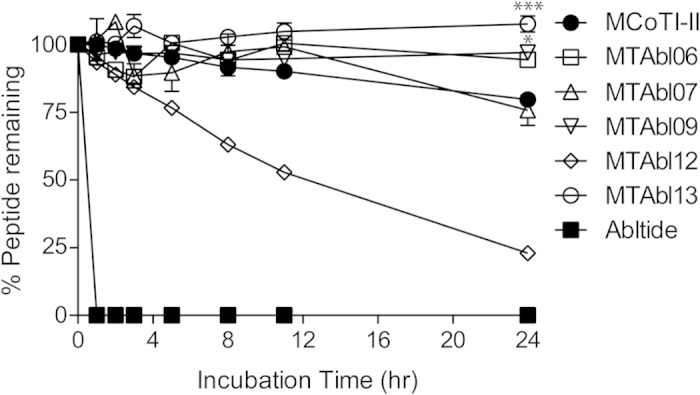
Stability of MTAbl peptides in human serum. Time course of the percentage of remaining peptide when incubated in 100% human serum at 37 °C for up to 24 h. The percentage of remaining MTAbl peptides was compared to that of MCoTI-II at 24 h time point using one-way ANOVA. MTAbl09 and MTAbl13 are significantly more stable than MCoTI-II (*p < 0.05 and ***p < 0.001, respectively) after being incubated in human serum for 24 h. Results are the mean ± SEM from three replicates.

**Figure 6:**
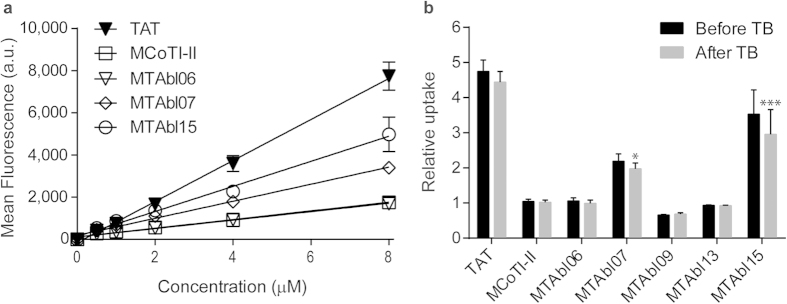
Internalization of MCoTI-II, MTAbl06, MTAbl07, MTAbl09, MTAbl13, and MTAbl15 into HeLa cells. (**a**) Peptides were conjugated with one Alexa Fluor® 488 molecule. The mean fluorescence intensity (a.u.) of HeLa cells treated with peptides at varying concentrations for 1 h at 37 °C was analyzed using a flow cytometer with excitation at 488 nm and emission at 530 nm (with 30 nm band pass). The cell-penetrating peptide TAT was included as a positive control. The values plotted in this figure were obtained after the addition of the aqueous soluble quencher trypan blue (TB), which quenches the fluorescence of non-internalized peptides and of cells that have their membranes compromised. (**b**) The relative cellular uptake to MCoTI-II of cells treated with TAT or five grafted peptides before and after the addition of TB. Internalization of MTAbl peptides into HeLa cells was compared to that of MCoTI-II by comparison of mean fluorescence emission intensity after the addition of TB using one-way ANOVA (*p < 0.05; ***p < 0.001). Results shown here are the mean ± SEM from three independent experiments.

**Figure 7:**
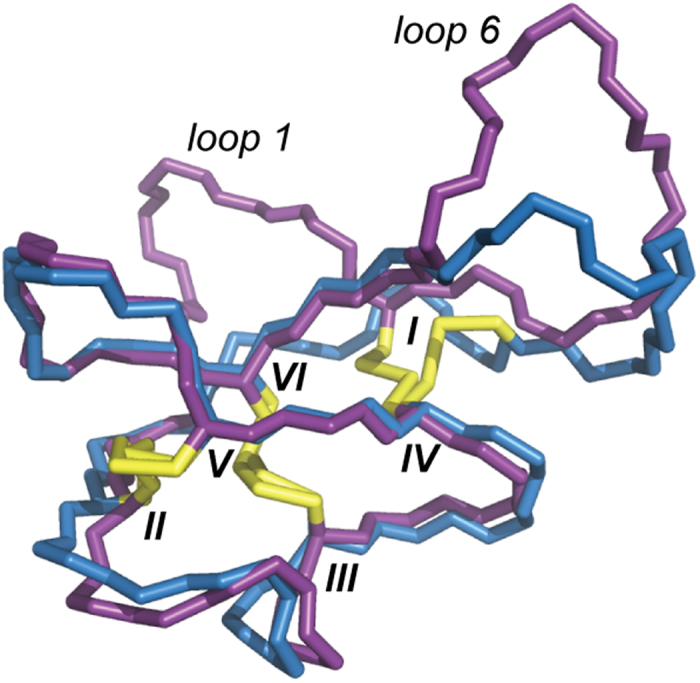
Superimposition of the NMR solution structures of MCoTI-II and of MTAbl13. Backbones of MCoTI-II (blue, PDB ID 2ib9) and MTAbl13 (purple) are shown using stick representation. Disulfide bonds that form the cystine knot motif are in yellow sticks.

MTAbl13 contained a graft in two of its loops and had the most potent inhibitory activity of all MTAbl peptides; therefore, its tertiary structure in solution was elucidated using NMR spectroscopy. Distance and dihedral angle restraints were derived from one- and two-dimensional homonuclear ^1^H NMR experiments and used in structure calculations (Supplementary Table S2 and Supplementary Fig. S7). The refinement statistics are shown in Supplementary Table S2 and the structure coordinates have been deposited in the Protein Data Bank (PDB ID: 2mt8). As illustrated in Fig. 7, loops 2, 3, 4 and 5 of MTAbl13 are similar in conformation to the corresponding loops of MCoTI-II, in agreement with the secondary Hα chemical shift analysis presented in Fig. 3. The disulfide bond that connects loops 1 and 6 was found to adopt a different conformation to that of MCoTI-II. This change might result in a small change of conformation of the grafted abltide when at the interface with Abl kinase, and we hypothesize that it might be responsible for the observed improved activity compared to singly-grafted peptides, MTAbl06 and MTAbl09.

The secondary Hα chemical shift analysis suggested that both loops 1 and 6 of MTAbl13 are generally disordered. A superimposition of the 20 lowest energy conformations resulting from the structure calculations further suggested that loops 1 and 6 are flexible compared to the rest of the molecule (Supplementary Fig. S8). Loop 1 adopts a more constrained conformation compared to loop 6 (Supplementary Fig. S8), but both loops protrude away from the cystine knot core, and could presumably insert into the substrate-binding pocket of Abl kinase. None of the NMR models of the more constrained conformation of loop 1 corresponded to the kinase-bound conformation. By contrast, loop 6 was more flexible and therefore might be able to adopt the bound conformation.

### Cell viability assay against K562 cells

The effect of MTAbl peptides on the growth and apoptosis of K562 cells was evaluated using a resazurin-based cell viability assay. K562 is an immortalized cell line derived from a 53-year-old female CML patient in blast crisis^38^ and has been used extensively as a model for CML studies. Although MTAbl peptides showed potent activity against Abl kinase *in vitro*, MTAbl06, MTAbl07, MTAbl13 and MTAbl15 did not inhibit the growth of K562 up to 64 μM (Supplementary Table S1).

## Discussion

Although current frontline therapies are initially effective at inhibiting cancer progression, resistance can develop after a few years of continuous treatment because of mutations in the ATP binding site that render these drugs ineffective. All human kinases share similar ATP binding pockets, and designing drugs targeting these sites with high specificity is difficult. The ligand-binding sites of kinases, on the other hand, are more distinct and less prone to mutations than the ATP binding cleft because of the functional requirement to target specific substrates^39^,^40^, suggesting that substrate-based inhibitors have the potential to be developed as TKIs with high specificity.

We attempted to turn abltide from being a substrate into an inhibitor by replacing the phosphorylatable tyrosine with natural or non-natural amino acids, but none of the resulting peptides were active. Phosphorylated abltide (p-abltide) was then shown to be as active as abltide using the BacKin inhibition assay, suggesting that the substrates of Abl kinase can serve as inhibitors after they have been phosphorylated. Similarly, the phosphorylated MTAbl peptides showed similar activity as their non-phosphorylated form, *i.e*. MTAbl12 *vs* MTAbl06 and MTAbl14 *vs* MTAbl13. Some aspects of the inhibition of Abl kinase by phosphorylated substrates remain unclear; for example, the kinase state to which the phosphorylated peptides bind is unknown. Indeed, phosphorylated peptides cannot possibly bind to the inactive conformational state of Abl kinase because the activation loop adopts a conformation incompatible with substrate binding. As the charged phosphate group of the phosphorylated tyrosine would create both steric and charge repulsion with the ATP molecule, an alternative state of the kinase in which the activation loop is in an active form but the ATP cleft is empty or occupied by an ADP molecule is possibly the state targeted by the phosphorylated peptides.

In total, 14 synthetic cyclic peptides incorporating one or two grafted abltides were designed and generally showed medium to high inhibitory activity against Abl kinase. The most potent peptides, *i.e*. MTAbl06, MTAbl07 and MTAbl13, exhibited low micromolar IC_50_ values, as tested using the BacKin assay. The IC_50_ values determined using this assay potentially underestimate the ability of the MTAbl peptides to inhibit Abl kinase, as these values are measured based on a competition with abltide, which is the optimal substrate for Abl kinase and has higher affinity for Abl than its physiological substrates. Indeed, the Michaelis-Menten constant K_m_ for phosphorylation of abltide by Abl is between 4 μM^20^ and 21 μM^41^, but the phosphorylation of the peptide with flanking sequences of the phosphorylatable tyrosine (Tyr-207) derived from the canonical substrate CRKL has a K_m_ of 134 μM^41^. The K_m_ of abltide is of the same order of magnitude as the best IC_50_ values of MTAbl peptides, indicating that the corresponding designed peptides have probably reached the maximal potential of abltide. Molecular modeling studies suggested that the relative potencies of the MTAbl peptides were mainly dependent on the ability of the grafted abltides to adopt an optimal conformation despite the scaffold displaying a different orientation relative to the kinase. This result indicates that the native MCoTI-II scaffold does not contribute significantly to the affinity with the kinase.

MTAbl peptides grafted either in loop 1, *e.g*. MTAbl09, or in loop 6, *e.g*. MTAbl06, resulted in potent inhibition of Abl kinase. Additional basic residues at the C-terminus of the graft, *e.g*. MTAbl06 or MTAbl07, were also shown to slightly increase the kinase inhibition activity. Introducing abltide-derived sequences into both loops 1 and 6 in MTAbl13 increased inhibitory activity by 14-fold and 3-fold compared to the corresponding single grafted peptides MTAbl09 and MTAbl06, respectively. The solution structure of MTAbl13 by NMR shows a small relative displacement of loops 1 and 6 anchor cysteines compared to MCoTI-II and by extension presumably compared to the single-grafted analogs. This displacement could result in different presentation of the grafted peptides to the kinase. If both of the grafted abltides remain active, then an increase of local concentration is a possible explanation of the improved activity, as it has been proposed by another recent study where grafting two copies of the same active peptide in a backbone-cyclic scaffold also led to an increase in activity^42^.

MTAbl13 displayed inhibitory activity against both wild-type and [T315I]Abl kinase. Nevertheless, the IC_50_ of MTAbl13 against [T315I]Abl was 9-fold higher than that of Abl kinase. Although MTAbl13 exhibits decreased potency against [T315I] mutant compared to wild-type kinase, IM shows no effect on the [T315I] mutant up to 256 μM, which is >800-fold when compared to its low IC_50_ value towards wild-type kinase. The order of magnitude difference in inhibition activity of MTAbl13 against wild-type and mutant kinase might be due to the reported decreased kinase activity of [T315I]Abl towards classical BCR-ABL substrates^43^.

The stability of MTAbl peptides in human serum was evaluated to provide a guide to their potential stability *in vivo*. The time course studies indicated that the stability of MTAbl peptides is comparable to or higher than MCoTI-II. Interestingly, MTAbl07 is less stable than MTAbl06 despite high sequence similarity, and the slightly decreased stability of MTAbl07 might originate from the two additional basic residues (Arg) included in loop 6. A significant increase in serum stability of MTAbl13 (p < 0.001) compared to MCoTI-II after 24 h incubation was observed, and could be explained by fewer positively charged residues in MTAbl13 compared to MCoTI-II (Supplementary Table S1). Overall, the results clearly indicate that the cyclic cystine knot motif endow MTAbl peptides with exceptional enzymatic stability (half-life >24 h) despite the sequence variation.

The cellular uptake efficiency of substrate-based inhibitors show that A488-MTAbl07 internalizes into HeLa cells with a 2.2-fold increase in efficiency compared to A488-MCoTI-II. This finding is in accordance with previous studies demonstrating that the cellular uptake of MCoTI-II can be enhanced by increasing the overall positive charge of the peptide^44^,^45^. A488-MTAbl15, an analog of A488-MTAbl07 with the first and second Lys residues in loop 1 replaced with Arg, has a 3.5-fold higher cellular uptake than A488-MCoTI-II, which demonstrates that the cell internalization of MCoTI-II scaffold can be further improved by Lys to Arg substitution in loop 1 of the molecule.

Despite having high inhibitory activity against Abl kinase, the grafted peptides MTAbl06, MTAbl07, MTAbl13, and MTAbl15 showed no significant effect on the growth of K562 cells when administered at concentrations up to 64 μM. A possible explanation for their unexpectedly low potency against K562 cells is that only a limited amount is able to reach BCR-ABL within the cytoplasm. Although we showed that the grafted peptides can penetrate cells and, in fact, MTAbl07 and MTAbl15 have improved internalization efficiencies compared with MCoTI-II, a certain amount of the grafted peptides might have been trapped in cell compartments (e.g. endosomes). Our future work will focus on improving the amount of peptide reaching the cytoplasm by optimizing the cell internalization of the MTAbl peptides and thereby increase their effective concentration at the interface of protein-protein interactions involved in BCR-ABL signaling network. The molecular dynamics simulation studies suggest potential strategies to increase inhibitory activity by creating specific interactions between the MCoTI-II scaffold and Abl kinase and by trapping the grafted abltide into a conformation compatible with binding to Abl kinase. Whether the substrate-based Abl kinase inhibitors designed in this study can actively inhibit the BCR-ABL kinase, wild-type or with T315I mutation in cytoplasm remains to be determined; however, the kinase inhibitory activity combined with serum stability and improved cell uptake efficiency make these peptides promising candidates as the next-generation TKIs.

## Methods

### Isolation of MCoTI-II from *M. cochinchinensis*

Native MCoTI-II was isolated from *M. cochinchinensis* seed extract prepared as described previously^26^.

### Molecular modeling

An initial molecular model of abltide bound to active Abl kinase with an ATP molecule and two magnesium ions bound in the ATP-cleft was built by homology and refined using 50 ns MD simulations. Models of complexes between grafted cyclotides and Abl kinase were generated using an anchor based approach in Modeller and were refined using 20 ns MD simulations. An initial molecular model of abltide bound to active (DFG-in) Abl kinase with an ATP molecule and two magnesium ions bound in the ATP-cleft was built by homology on the basis of three templates: the crystal structures of inactive (DFG-out) Abl kinase bound to a chemically modified abltide (PDB ID 2g2f), the crystal structure of active (DFG-in) Abl kinase bound to an ADP molecule (PDB ID 2g2i) and the crystal structure of an active cAMP kinase bound to ATP and two magnesium ions (PDB ID 1atp). ProPKA^46^ was then used to compute the protonation states of all side chains and termini. Three-dimensional-RISM^47^,^48^ as implemented in AmberTools 13^49^ and placevent^50^ were employed to compute the positions of bound water molecules. Only water molecules with an accessible surface area below 20 A^2^, as measured with NACCESS (V2.1.1)^51^ were kept. This system was then placed in a dodecahedral box and solvated with a total of ~20,000 SPC water molecules. This system was energy minimized using 10,000 steps of steepest descent algorithm and water molecules were equilibrated over 1 ns molecular dynamics simulations with non-water molecules strongly restrained to their initial position. The system was then submitted to 50 ns molecular dynamics simulations carried out in triplicates using the gromos 54a7 forcefield^52^, the reaction-field electrostatic with a relative dielectric permittivity *ε* of 61^53^, the v-rescale temperature coupling^54^ set at 300 K, a Berendsen pressure coupling set at 1 atm^55^, a Coulombic radius of 1.4 Å, a Coulombic radius switch of 0.8 Å, and a Verlet cutoff scheme to compute Van der Waals interactions. All bonds were constrained with the LINCS algorithm^56^. Energy minimization and molecular dynamics simulations were carried out with Gromacs 4.6.5^57^. The three simulations were stable over the last 40 ns and converged toward a similar conformation. The final model and the NMR solution structure of MCoTI-II (PDB ID: 1ib9) were then used as a template to build using Modeller 9v12 the models of Abl kinase bound to MTAbl peptides (MTAbl00, MTAbl01, MTAbl02, MTAbl03, MTAbl08, MTAbl09, MTAbl10, MTAbl11) using the bound peptide as an anchor. The eight complexes were then prepared and studied by 20 ns molecular dynamics simulations using the same molecular modeling protocol described for the linear abltide/Abl system. The molecular dynamics simulations were analyzed using VMD^58^ and the gromacs tools. The electrostatic potential generated by Abl kinase was computed using the APBS software^59^.

### Peptide synthesis

Linear precursors of 14 MTAbl peptides containing an N-terminal Cys residue were synthesized manually using solid-phase peptide synthesis with Boc chemistry, as described previously^28^. In brief, precursor peptides were constructed on PAM-Gly-Boc resin via S-tritylmercaptopropionic acid as a thioester linker and cleaved from the resin with hydrogen fluoride. Through a thia zip reaction, the linear precursor peptides with multiple Cys and a C-terminal thioester form an α-amino thiolactone after a series of intramolecular acyl rearrangements and eventually undergo an irreversible *S,N* acyl migration to form an amide bond between the two ends^60^,^61^. Cyclization and oxidation of the MCoTI-II mutants was carried out in 0.1 M ammonium bicarbonate (pH 8.2) at 0.1 mg/mL peptide concentration. The peptide mixture was stirred at room temperature for 24 h and purified by reversed-phase high-performance liquid chromatography (RP-HPLC) on a preparative Phenomenex C_18_ column. A 1%/min gradient of solvent B (90% v/v acetonitrile/10% v/v H_2_O/0.045% v/v TFA (trifluoroacetic acid)) against solvent A (100% v/v H_2_O/0.05% v/v TFA) on RP-HPLC was used to separate peptides.

Abltide, [Y4A]abltide, [Y4F]abltide, and p-abltide containing a phosphorylated tyrosine at the fourth position were synthesized along with 15 other abltide analogs (abltide1–15), with the same position substituted with phenylalanine derivatives, as illustrated in Supplementary Fig. S5. The Phe derivatives used in this study were: **1**. 4-fluoro-L-phenylalanine, **2**. 4-chloro-L-phenylalanine, **3**. 4-methyl-L-phenylalanine, **4**. 4-nitro-L-phenylalanine, **5**. 4-amino-L-phenylalanine, **6**. L-homophenylalanine, **7.** 3,4-difluoro-L-phenylalanine, **8**. 3,4-dimethoxy-L-phenylalanine, **9**. pentafluoro-L-phenylalanine, **10**. *p*-trifluoromethyl-L-phenylalanine, **11**. 4-benzoyl-L-phenylalanine, **12**. *p*-phenyl-L-phenylalanine, **13**. 4-iodo-L-phenylalanine, **14**. 4-cyano-L-phenylalanine, and **15**. 4-*tert*-butyl-L-phenylalanine. Abltide and its 18 analogs were synthesized using solid-phase peptide synthesis with Fmoc chemistry on an automatic peptide synthesizer (Symphony^®^, Protein Technologies). Briefly, peptide chains were assembled on 2-chlorotrityl chloride (2-CTC) resin by consecutive addition of Fmoc-protected amino acids. Peptides were then removed from the resin by the treatment with TFA/triisopropylsilane/H_2_O (95:2.5:2.5, v/v) for 2 h. TFA was removed using rotary evaporation from the mixture and the residual solution was mixed with cold diethyl ether and solvent A/B (1:1, v/v). The aqueous layer containing crude linear peptides was collected and purified using RP-HPLC on a preparative C_18_ column to 95% purity. The mass of the peptides was confirmed with ESI-MS and the purity was examined by analytical HPLC.

### Detection of abltide phosphorylation by LC/MS

The time-course of phosphorylation of 30 μM abltide by 0.2 U/mL human active Abl kinase (1067 U/mg, Merck Millipore) and [T315I]Abl (Abl(T315I) protein, Merck Millipore) was analyzed using Liquid Chromatography-Mass Spectrometry (LC/MS, Shimadzu LCMS-2020). Abltide and Abl kinase were prepared and mixed in kinase buffer (50 mM Tris-HCl, 10 mM MgCl_2_, 0.1 mM EDTA, 2 mM dithiothreitol and 0.01% Brij 35 (v/v), pH 7.5; NEBuffer for protein kinases, New England Biolabs) at a desired concentration and the reaction was initiated by the addition of 500 μM ATP (adenosine 5′-triphosphate disodium salt hydrate, Sigma-Aldrich). The mixture was incubated at 37 °C with gentle shaking and the reaction was terminated with 10% 20 mg/mL dihydroxybenzoic acid at nine time points (0, 5, 10, 20, 30, 45, 60, 90 and 120 min). Samples were injected onto a C_18_ analytical column (Jupiter 300 5 μm 300 Å, 150 × 2.0 mm, Phenomex) and the mass of each component was evaluated using MS. The percentage of phosphorylation of abltide was determined by comparing the peak area of phosphorylated abltide (1417 Da) with the peak area of 30 μM abltide from LC (1336.6 Da). Triplicate experiments were conducted on three independent days.

### BacKin assay

BacKin, an approach to evaluate the inhibition of Abl kinase activity by using bacterial surface displayed substrate, was used as described previously^33^. Briefly, *E. coli* (MC1061) cells with the abltide sequence displayed on the surface (abltide-expressing cells) were subcultured and grown at 37 °C with constant shaking at 225 rpm for 2 h or until the optical density of the culture at 600 nm reached 0.6. The culture was induced with 0.04% (w/v) l-arabinose and incubated at 37 °C for an additional hour. Abltide-expressing cells were then spun down at 3000 *g* for 4 min, washed with cold phosphate buffered solution (PBS) and resuspended in 0.2 U/mL of Abl kinase or [T315I]Abl prepared in kinase buffer (NEBuffer, New England Biolabs). Inhibition of the Abl kinase activity was evaluated by incubating abltide-expressing cells with designed peptides at concentrations ranging from 64 μM to 0.5 μM, followed by the addition of 500 μM ATP. Assays were carried out in duplicate at 37 °C on a shaker at 180 rpm for 30 min. The reactions were terminated by centrifugation at 3000 *g* for 4 min at 4 °C. Cells were washed with ice-cold PBS twice before incubating with 1 μg/mL biotinylated antibody anti-phosphotyrosine 4G10 (Merck Millipore) at 4 °C on shaker for 45 min. Cells were spun down and the supernatant containing antibody was removed before the addition of 5 μg/mL streptavidin-R-phycoerythrin (SAPE, Invitrogen). Cells were incubated for an additional 45 min at 4 °C with the subsequent removal of residual SAPE. Flow cytometry (BD Canto II) was used to monitor the fluorescence intensity of each sample using 488 nm excitation and the 530/30 bandpass filter.

### One- and two-dimensional 1H NMR

The correct folding of MTAbl peptides was confirmed using ^1^H NMR. Peptides were dissolved in H_2_O/D_2_O (9:1, v/v) to a concentration of 1–2 mg/mL. NMR spectra were recorded on a Bruker Avance-600 MHz NMR spectrometer at 298 K. The mixing time was 80 ms and 100–300 ms for TOCSY and NOESY experiments, respectively. Spectra were analyzed using Sparky. Spectra were internally referenced to 2,2-dimethyl-2-silapentane-5-sulfonic acid (DSS) at 0.00 ppm.

### Structure determination of MTAbl13

Structure calculations were carried out for MTAbl13 as described previously for other cyclic disulfide-rich peptides^42^. Briefly, samples of MTAbl13 were prepared in either 90% H_2_O/10% D_2_O (9:1, v/v) or 99.96% D2O (Cambridge Isotope Laboratories, Inc.) at ~1 mM and pH ~3. One- and two-dimensional NMR spectra (^1^H, ^1^H TOCSY, NOESY, DQF-COSY and ECOSY, and ^1^H, ^13^C HSQC) were acquired using Avance-500 or Avance-600 MHz spectrometers (Bruker). Spectra were processed with TopSpin (Bruker) and analyzed using CCPNMR^62^. Inter-proton distance constraints were calculated from the relative intensities of NOE cross-peaks. ^3^J_HN-Hα_ coupling constants were measured from one-dimensional spectra or from anti-phase cross-peak splitting in the DQF-COSY spectrum, and ^3^J_Hα-Hβ_ coupling constants were measured from the ECOSY spectrum. Backbone amide protons involved in intramolecular hydrogen bonds were identified by their temperature sensitivities and deuterium exchange rates (Supplementary Fig. 7). Preliminary structure calculations were performed using CYANA 3.0^63^, followed by further structure calculations and refinement in a water shell using CNS^64^. An ensemble of 20 structures with the lowest energy was selected and the refinement statistics are presented in Supplementary Table S2.

### Serum stability assay

The stability of abltide-grafted analogs MTAbl06, MTAbl07, MTAbl12, MTAbl13 and native MCoTI-II was assessed in 100% human male AB serum (Sigma) using a method modified from a previous study^27^. All peptides were tested at a final concentration of 30 μM in 100% human serum. Peptides were incubated in human serum at 37 °C for 0, 1, 2, 3, 5, 8, 11, and 24 h. Reactions were stopped at the stipulated times and the serum proteins in each sample were denatured and precipitated, followed by centrifugation at 17,000 *g* for 10 min to separate the serum proteins from peptide samples. One hundred μL of supernatant was loaded on an analytical column (150 × 2.0 mm) and run on RP-HPLC using a linear 1%/min gradient of 0–40% solvent B at a 0.3 mL/min flow rate. Equivalent amount of peptides were incubated with PBS and processed in parallel with serum-treated samples at 0 and 24 h time points as negative controls. The elution time for each peptide was determined by the PBS control from 0 time point. The percentage of peptide remaining at each time point was calculated using the height of the serum-treated peptide peak from time 0 as 100% recovery.

### Labeling of MCoTI-II and analogs with fluorophore

Native MCoTI-II, MTAbl06, MTAbl07, MTAbl09, MTAbl13 and abltide were labeled with Alexa Fluor® 488 (abbreviated as A488) using a method described by Cascales *et al*.^26^. In brief, peptide solutions prepared in 0.1 M sodium bicarbonate (pH 8.3) were incubated with 2-fold excess (molar ratio) of Alexa Fluor® 488 5-SDP ester (Molecular Probes, Life technologies) for 2 h at room temperature on a roller mixer. The conjugated peptides were purified using RP-HPLC and the mass was confirmed by ESI-MS. Peptides labeled with one A488 molecule were collected and used in cellular uptake studies. TAT (NH_2_-YGRKKRRQRRRPPQG-COOH) was labeled with the same fluorophore and used as the positive control in internalization assays.

### Cell culture

Human cervical cancer cells (HeLa) were seeded in 175-cm^2^ tissue culture flasks (Falcon), in *Dulbecco’s Modified Eagle’s Medium (*DMEM) supplemented with 10% fetal bovine serum, 100 U/mL penicillin and 100 mg/mL streptomycin, until 80% confluent. K562 cells were cultured in Roswell Park Memorial Institute (RPMI) 1640 medium supplemented with 2 mM l-glutamine, 10% fetal bovine serum, 100 U/mL penicillin, and 100 mg/mL streptomycin and grown to 80% confluence.

### Mammalian cell viability assay

Cytotoxicity of the MTAbl peptides towards HeLa cells was evaluated before the cell internalization assay. The mammalian cell cytotoxicity assay was conducted using a method described earlier^65^. HeLa cells were seeded the day before the assay in 96-well tissue culture-treated plates (2.5 × 10^3^ cells/well). Peptides TAT, MCoTI-II, MTAbl06, 07, 09, 13 and 15 were dissolved and diluted in sterile H_2_O (2-fold dilutions starting from 640 μM). Peptide solutions were diluted 10-fold with serum-free medium and incubated with cells in triplicates for 2 h. Controls with H_2_O or 0.01% (v/v) Triton X were included to have 100% and 0% cell viability, respectively. Following incubation the peptide solutions were removed and aliquots of 10 μL of 0.02% (w/v) sterile resazurin (resazurin sodium salt, Sigma) were added to each well containing 100 μL of fresh medium. Cells were incubated under standard conditions of 37 °C and 5% CO_2_ for 22 h. The absorbance of the plates was measured on a plate reader at 540 and 620 nm. The cytotoxicity of peptides abltide, MCoTI-II, MTAbl06, 07, 13 and 15 against K562 cells was evaluated using the resazurin-based assay with 5 × 10^3^ cells per well.

### Evaluation of cellular uptake of the mutant MTAbl peptides by FACS

The cellular uptake of the fluorescently-labeled peptides was examined in HeLa cells using a method described previously^44^. In brief, 10^5^ cells/well were seeded in a 24-well plate and incubated at 37 °C in a humidified atmosphere (5% CO_2_) overnight. Peptides labeled with one Alexa Fluor® 488 were dissolved in sterile water by 2-fold serial dilutions starting at 80 μM. 100 μL of serum-free medium in the absence or presence of labeled peptides with final concentrations at 0.5, 1, 2, 4, or 8 μM was added in each well and incubated with cells for 1 h at 37 °C. After 1 h incubation, peptides were removed and cells were rinsed with Dulbecco’s Phosphate-Buffered Saline (DPBS, Life Technologies). Following that, cells in 24-well plate were treated with 0.25% Trypsin-EDTA (Gibco^®^, Life technologies) for 3 min, washed off with DPBS and individually transferred to Eppendorf Tubes®. Cells were spun down at 500 *g* for 3 min to remove the supernatant and re-suspended with 1 mL of DPBS. The fluorescence intensity of cells excited at 488 nm was measured using FACS flow cytometry (BD Canto II) using a 530/30nm bandpass filter.

### Statistical analysis

Results are shown as the mean ± SEM from two or three replicates as indicated. Statistical significance was determined using one-way ANOVA with Bonferroni’s multiple-comparison test within groups.

## Additional Information

**How to cite this article**: Huang, Y.H. *et al*. Design of substrate-based BCR-ABL kinase inhibitors using the cyclotide scaffold. *Sci. Rep*. **5**, 12974; doi: 10.1038/srep12974 (2015).

## Supplementary Material

Supplementary Information
