# Supplementary material for: Design of substrate-based BCR-ABL kinase inhibitors using the cyclotide scaffold
Source: Sci Rep. 2015 Aug 12;5:12974. doi: 10.1038/srep12974 (PMC4532999; doi:10.1038/srep12974)

Supplementary Information

Design of substrate-based BCR-ABL kinase inhibitors using cyclotide scaffold

Yen-Hua Huang, Sónia T. Henriques, Conan K. Wang, Louise Thorstholm, Norelle L. Daly, Quentin Kaas and David J. Craik

Supplementary Table S1: Concentrations required to inhibit the phosphorylation of abltide catalyzed by Abl kinase (IC_{50}) using the BacKin assay, cytotoxicity against K562 cells and the net charge of MTAbl peptides. Data were fitted to a sigmoidal dose-response curve with variable slopes and analyzed with GraphPad Prism 6. Results shown here are the mean ± SEM from two independent experiments.

Compounds	IC_{50} (μM)		Net charge
	BacKin	K562	
IM	0.3 ± 0.03	–	–
abltide	5.7 ± 3.9	> 64	2
p-abltide	9.6 ± 0.9	–	2
MCoTI-II	> 64	> 64	3
MTAbl05	15.2 ± 7.0	–	4
MTAbl06	5.5 ± 1.6	> 64	4
MTAbl07	4.1 ± 0.6	> 64	6
MTAbl09	18.3 ± 7.6	–	0
MTAbl10	39.3 ± 4.2	–	0
MTAbl11	> 64	–	0
MTAbl12	4.7 ± 0.9	–	4
MTAbl13	1.3 ± 0.1	> 64	1
MTAbl14	2.6 ± 0.2	–	1
MTAbl15	–	> 64	6
MTAbl13[a]	12.2 ± 1.2	–	1
IM[a]	> 256	–	–

[a] *IC_{50} of compounds tested against [T315I] mutant.*

Supplementary Table S2: NMR refinement statistics for the 20 lowest energy structures of MTAbl13

**Distance constraints**	
Total NOE	396
Intra-residue	133
Inter-residue	263
Sequential ($\lvert i - j\rvert = 1$)	160
Medium range ($\lvert i - j\rvert \leq 4$)	43
Long-range ($\lvert i - j\rvert \geq 5$)	60
Hydrogen bonds	13
Disulfide bond restraints	3
Total dihedral angles	
ϕ	25
χ^1	12
Mean no. of NOE violations > 0.3 Å	0
Mean no. of dihedral violations > 3°	0
**Structural Statistics**	
Rmsd from mean structure	
Residues 10-28	
Backbone atoms (Å)	0.56 ± 0.21
Heavy atoms (Å)	1.48 ± 0.30
Loop 1 (Residues 2-9)	
Backbone atoms (Å)	0.49 ± 0.23

Heavy atoms (Å)	1.60 ± 0.41
Loop 6 (Residues 29-39)	
Backbone atoms (Å)	2.09 ± 0.57
Heavy atoms (Å)	3.97 ± 0.90
Stereochemical quality (excl. Gly and Pro)	
Ramachandran favoured/allowed (%, fraction of residues)	100, 600/600
Ramachandran outliers (%, fraction of residues)	0.0, 0/600

Supplementary Figure S1. Root mean square deviations of linear abltide/Abl complex over 50 ns molecular dynamics simulations from the first frame. The conformations in the initial frame is the one displayed in the crystal structure of abltide conjugated to an ATP-like molecule and bound to Abl kinase (PDB ID: 2g2f).

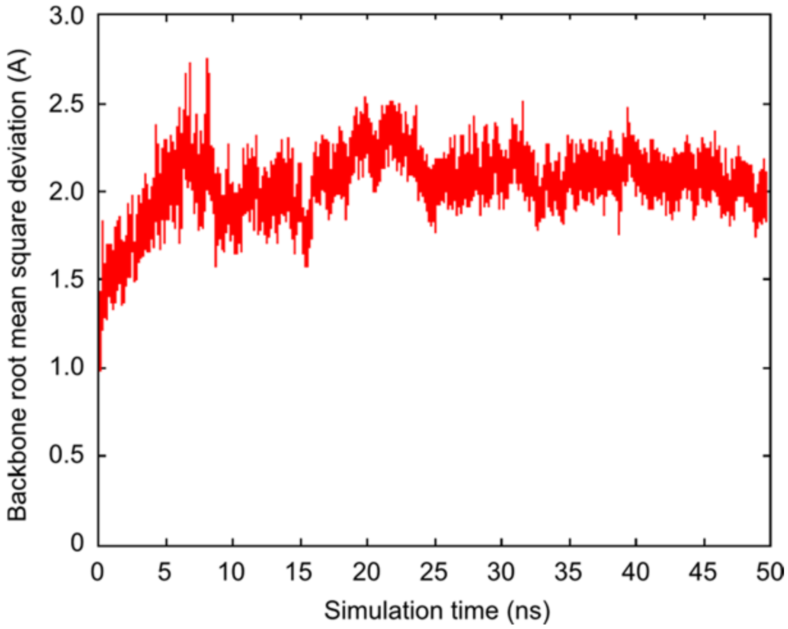

Supplementary Figure S2. Model of the interaction between Abl kinase and MTAbl00, 01, 02, 03, 08, 09, 10 and 11. The models were refined using 20 ns molecular dynamics simulations. Abl kinase is represented in white cartoon, MTAbl peptides in blue cartoon, ATP molecule in orange sticks and magnesium ions as green spheres. The MTAbl peptide disulfide bonds and grafted epitope side chains are in stick representations.

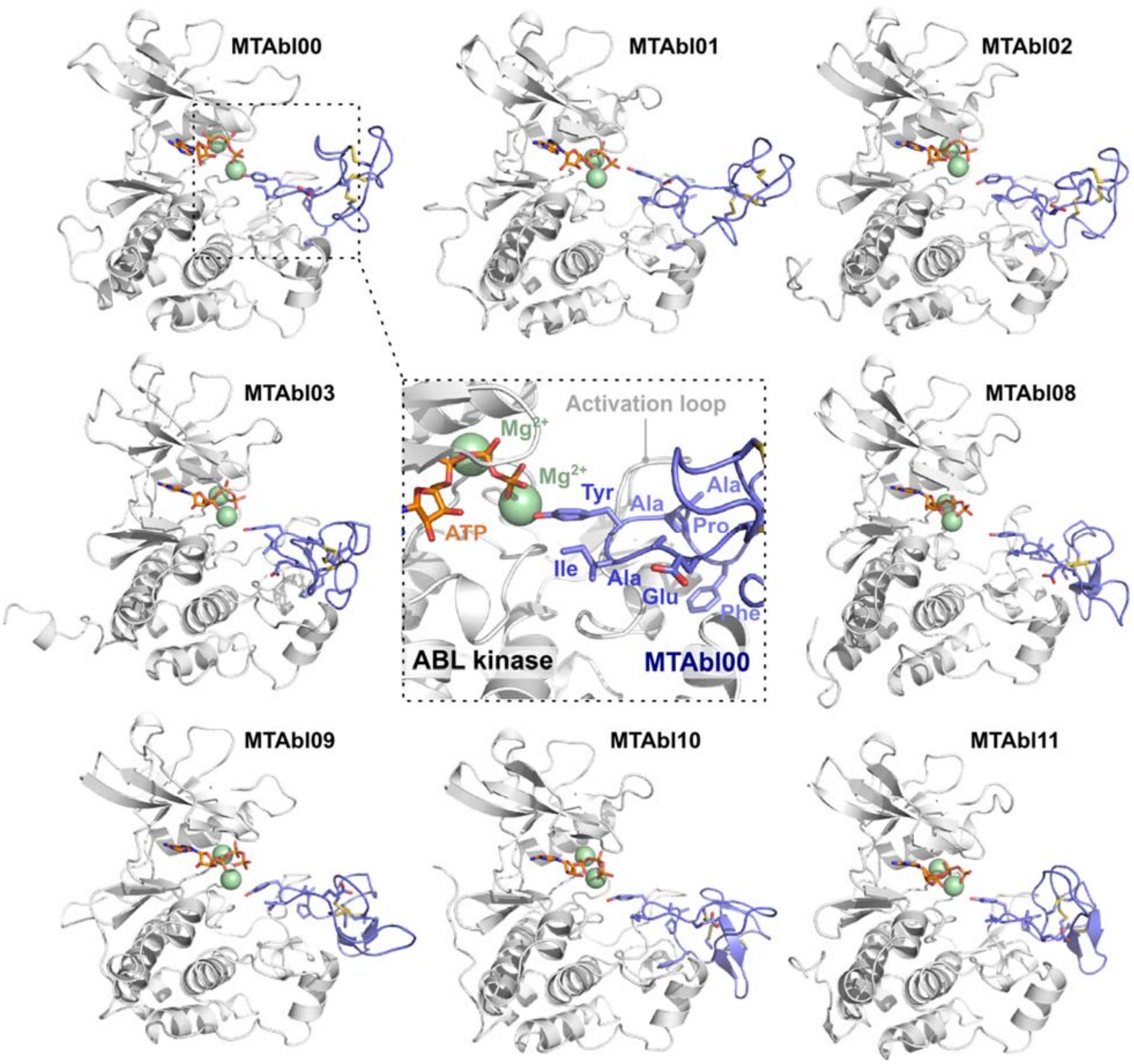

Supplementary Figure S3. Backbone root mean square deviation of Abl kinase for all MD simulations to the first frame of each simulation.

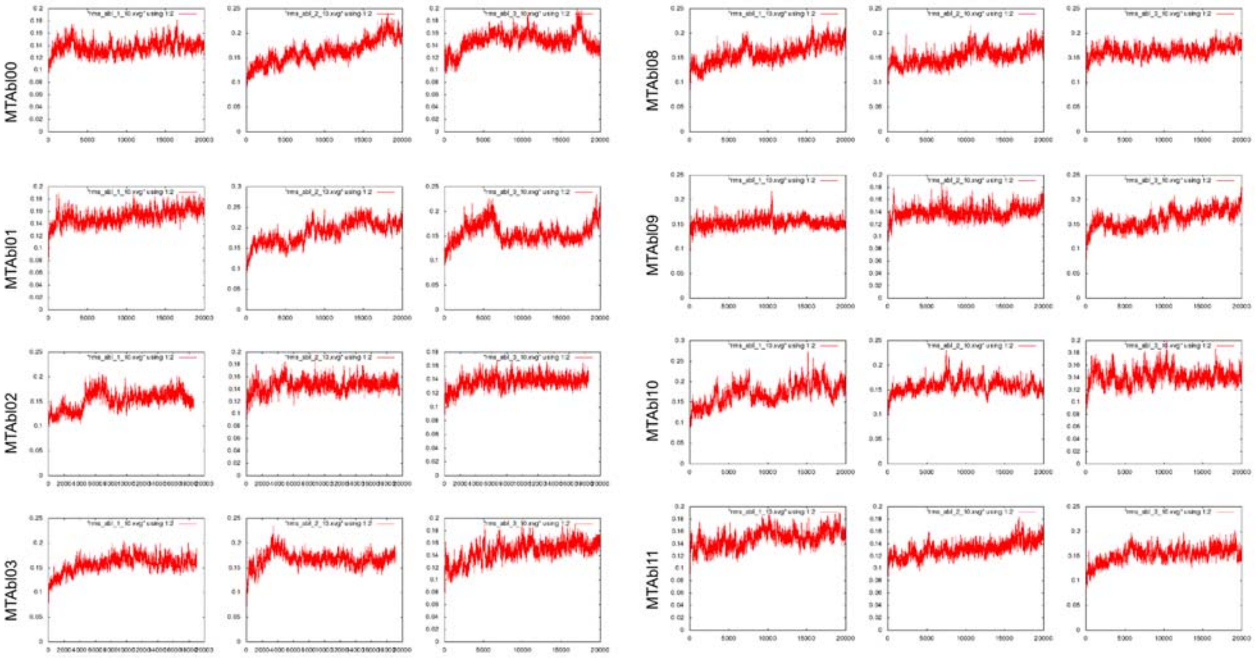

Supplementary Figure S4. The root mean square deviation of grafted sequences (graft rmsd) in MTAbl08, 09, 10 and 11. Graft rmsd is define as the backbone rmsd of the grafted peptides to the corresponding peptide segment displayed by linear abltide when in complex with Abl.

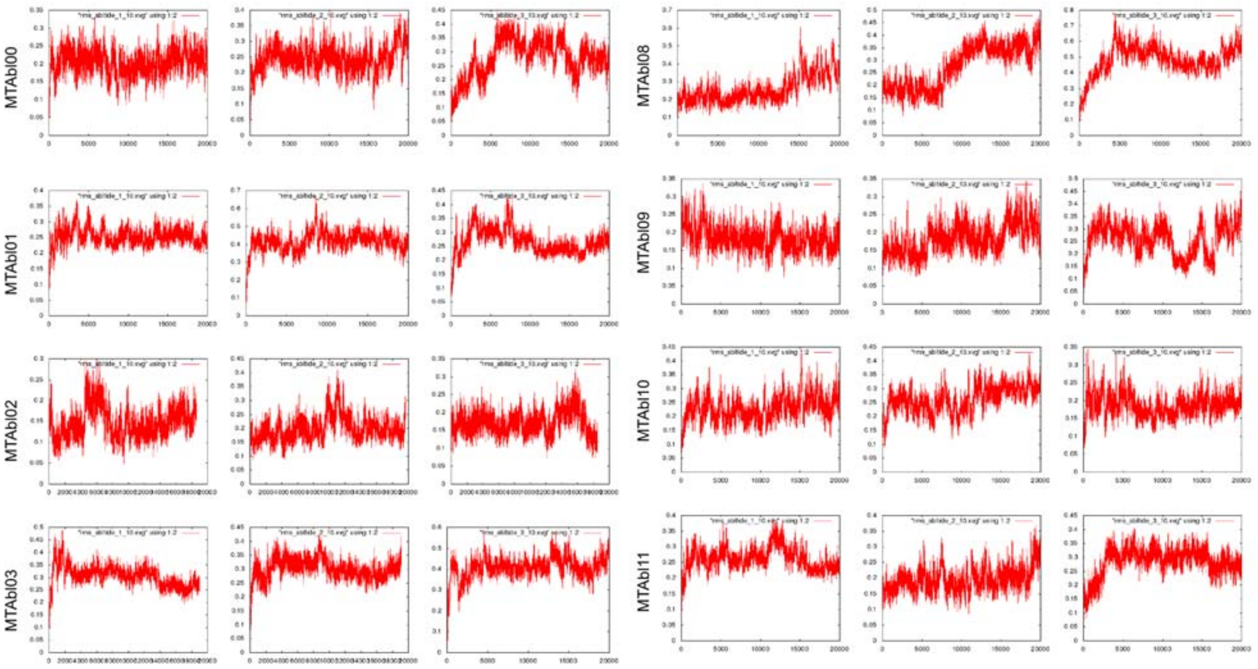

Supplementary Figure S5: The tyrosine in abltide sequence was substituted with phosphorylated tyrosine or phenylalanine derivatives. **1**. 4-fluoro-L-phenylalanine, **2**. 4-chloro-L-phenylalanine, **3**. 4-methyl-L-phenylalanine, **4**. 4-nitro-L-phenylalanine, **5**. 4-amino-L-phenylalanine, **6**. L-homophenylalanine, **7**. 3,4-difluoro-L-phenylalanine, **8**. 3,4-dimethoxy-L-phenylalanine, **9**. pentafluoro-L-phenylalanine, **10**. *p*-trifluoromethyl-L-phenylalanine, **11**. 4-benzoyl-L-phenylalanine, **12**. *p*-phenyl-L-Phenylalanine, **13**. 4-iodo-L-phenylalanine, **14**. 4-cyano-L-phenylalanine, and **15**. 4-*tert*-butyl-L-phenylalanine. Mutant abltides with a single point mutation on position four were synthesized and the inhibition efficacy were evaluated using BacKin assay.

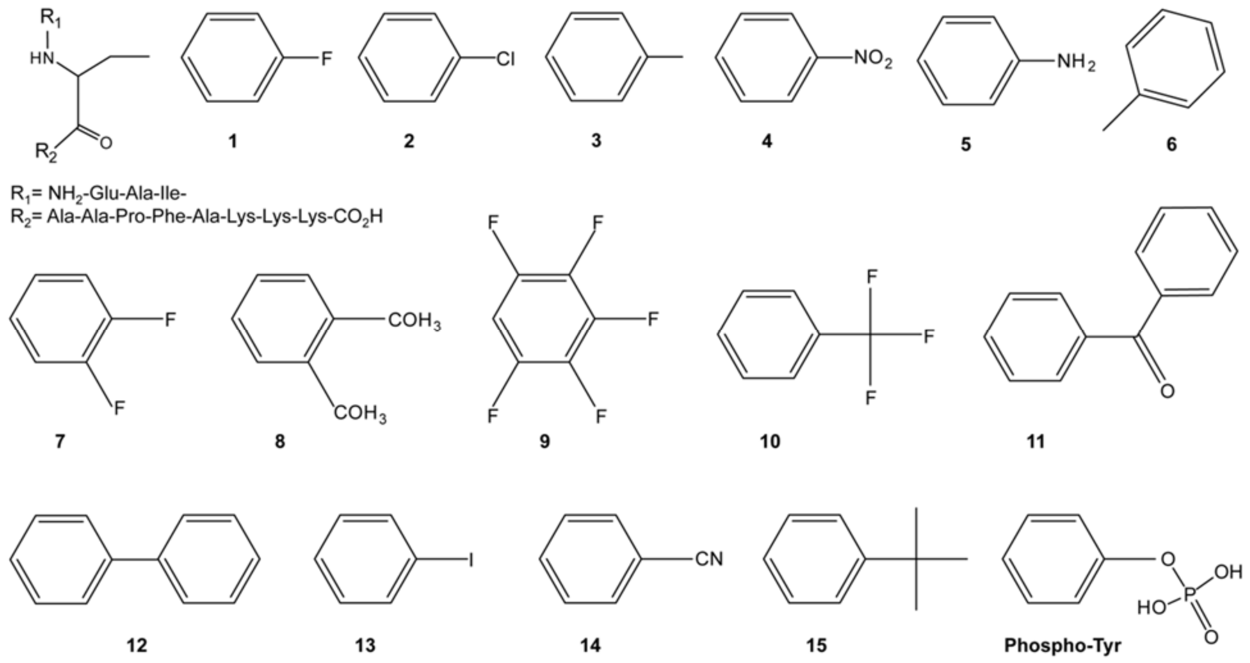

Supplementary Figure S6. Time-course of abltide phosphorylation catalyzed by 0.2 U/mL Abl (squares) or [T315I] kinase (triangles) in the presence of 500 μM ATP at 37ºC obtained with LC-MS analysis. The time required to phosphorylate half of the abltide molecules, $t_{1/2}$ ± SEM, was calculated by fitting using an equation for pseudo-first order association kinetics.

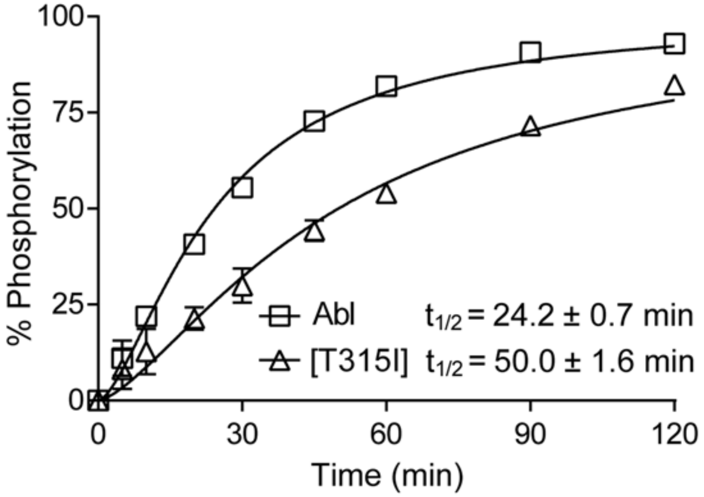

Supplementary Figure S7. Hydrogen bond properties of MTAbl13. Amide temperature coefficients ($\Delta\delta_{NH}/\Delta T$) of MTAbl13 were measured in 90% (v/v) H_2O, 10% (v/v) D_2O, pH ~3. The dotted line represents a $\Delta\delta_{NH}/\Delta T$ of -4.6 ppb/K, which has been proposed as a cut-off to predict whether amides are involved in intra-molecular hydrogen bonds. H/D exchange was also carried out on MTAbl13; open white circles represent slow-exchanging amides. The sequence of MTAbl13 aligned to MCoTI-II is shown at the bottom of the chart. Yellow circles mark the position of the cysteine residues. Shaded circles identify residues that have amides predicted to be involved in hydrogen bonding by the program MOLMOL using the solution structure of MCoTI-II (PDB ID: 1IB9).

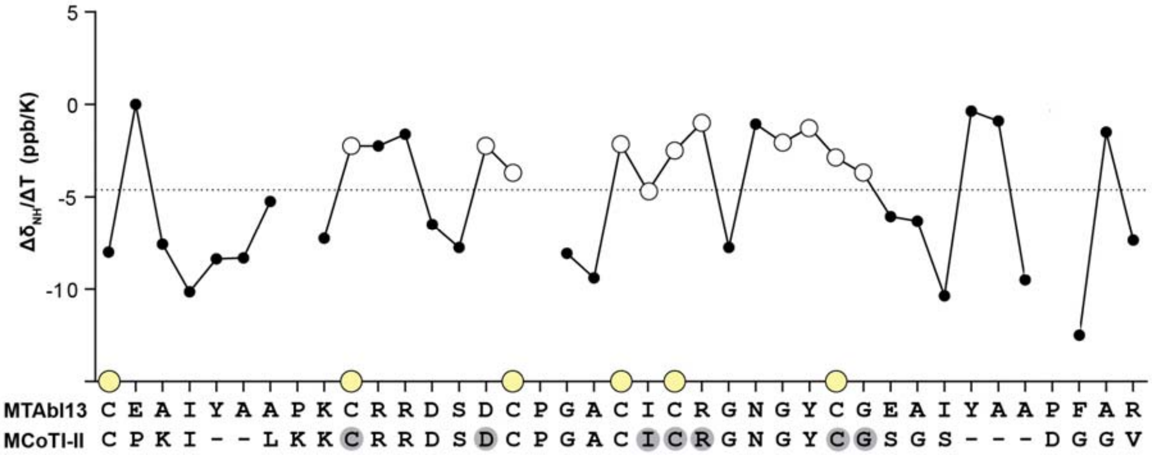

Supplementary Figure S8. Superposition of the 20 lowest energy structures of MTAbl13. Panel A shows the a superposition of the 20 lowest energy structures aligned over residues 10–19 (blue), which belong to the unmodified regions with respect to the parent peptide, MCoTI-II. Cysteine residues are labelled with Roman numerals and the two grafted loops are labelled and colored red or green. Panel B shows a structural alignment of loop 1 (red), whereas Panel C shows a structural alignment of loop 6 (green). Selected residues are labelled.

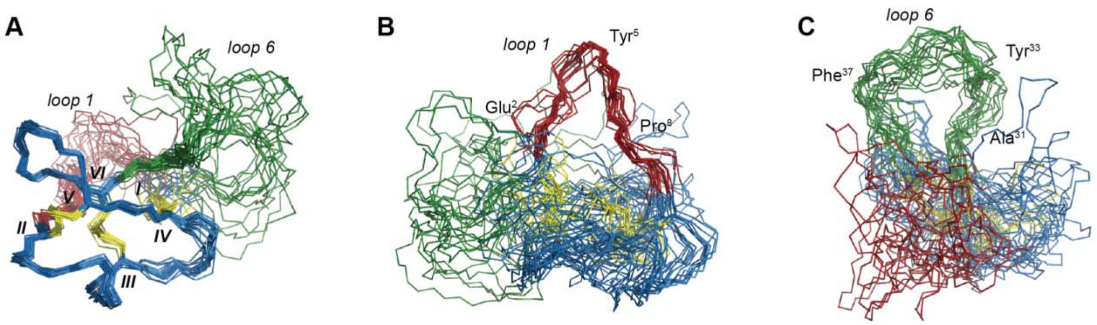

Supplement: Supplementary Information [file srep12974-s1.pdf]
